# Efficient and Controllable Synthesis of 1-Aminoanthraquinone via High-Temperature Ammonolysis Using Continuous-Flow Method

**DOI:** 10.3390/molecules28114314

**Published:** 2023-05-24

**Authors:** Feng Zhou, Lei Cai, Wenjie Ye, Kai Zhu, Jin Li, Yanxing Li, Weichuan Xu, Pan Wang, Chuansong Duanmu

**Affiliations:** 1 National & Local Joint Engineering Research Center for Deep Utilization Technology of Rock-Salt Resource, Faculty of Chemical Engineering, Huaiyin Institute of Technology, Huaian 223003, China; clei@hyit.edu.cn (L.C.);; 2 China Construction Industrial & Energy Engineering Group Co., Ltd., 6 Wenlan Road, Qixia District, Nanjing 210023, China

**Keywords:** ammonolysis, continuous-flow, 1-aminoanthraquinone, optimization, kinetic

## Abstract

Anthraquinone dyes are the second most important type of dyes after azo dyes. In particular, 1-aminoanthraquinone has been extensively utilized in the preparation of diverse anthraquinone dyes. This study employed a continuous-flow method to synthesize 1-aminoanthraquinone safely and efficiently through the ammonolysis of 1-nitroanthraquinone at high temperatures. Various conditions (reaction temperature, residence time, molar ratio of ammonia to 1-nitroanthraquinone (*M-ratio*), and water content) were investigated to explore the details of the ammonolysis reaction behavior. Operation conditions for the continuous-flow ammonolysis were optimized using Box–Behnken design in the response surface methodology, and ~88% yield of 1-aminoanthraquinone could be achieved with an *M-ratio* of 4.5 at 213 °C and 4.3 min. The developed process’s reliability was evaluated by performing a 4 h process stability test. The kinetic behavior for the preparation of 1-aminoanthraquinone was investigated under continuous-flow mode to guide the reactor design and to gain a deeper understanding of the ammonolysis process.

## 1. Introduction

Among dyes, anthraquinone dyes rank second only to azo dyes in terms of importance [1,2]. Anthraquinone dyes are usually used where extreme properties and color fastness or special colors are required, and vat dyes are composed of anthraquinones in most cases. Aminoanthraquinones, especially 1-amino and 1,5-diaminoanthraquinones, contribute to the preparation of a wide range of anthraquinone dyes. Furthermore, 1-aminoanthraquinones generally can be synthesized industrially using anthraquinone-1-sulfonic acid and 1-nitroanthraquinone as starting materials [2]. These two types of raw materials have been used to develop a variety of synthesis routes, but in industry, replacing the nitro groups in 1-nitroanthraquinone with ammonia is preferred because it offers significant advantages in terms of productivity, raw material availability, and cost-effectiveness. Nonetheless, such synthesis of 1-aminoanthraquinone is a typical ammonolysis reaction regulated strictly by local policies as a hazardous chemical process [3].

To realize expected conversion of the starting material within an economical reaction time, high-temperature and high-pressure operation are typically used in ammonolysis reactions, which are usually exothermic but have a low reaction rate. It is important to note that ammonia is a volatile substance, and the formed vapors in air may be explosive under normal conditions when concentrations are between 16 and 28 percent [4,5]. The system can easily reach its explosion limit once there is a leak in the reaction process under high temperatures and high pressures. A variety of initiatives have been undertaken to reduce safety risks associated with ammonolysis reactions, make them greener, and increase their efficiency. Specifically, researchers focus on developing diverse catalysis systems from a chemistry perspective as a means of enhancing the efficiency of the ammonolysis reaction conducted under a relatively mild reaction environment [6,7,8,9,10,11]. For example, a novel, efficient, and environmentally friendly nano-catalyst of a functionalized mesoporous material grafted with platinum was designed to synthesize anilines by ammonolysis of aryl halides with aqueous ammonia over a period of ten hours at room temperature [8]. As synthetic chemistry greatly benefits from flow chemistry, it is possible to anticipate that a strategy based on continuous-flow technology will improve the conventional ammonolysis process for synthesizing 1-aminoanthraquinone from 1-nitroanthraquinone from the perspective of process intensification. It has been extensively reviewed how continuous-flow microreactors offer enhanced heat and mass transfer properties, rigorous regulation of process parameters, an easy means of manipulating reaction conditions, and inherent safety [12,13,14,15,16,17]. By operating in the continuous-flow mode, the reaction system can be superheated without compromising safety, and it can be operated over a wider temperature range. Despite decades of industrial progress in 1-aminoanthraquinone preparation, batch reactions at high temperatures and pressures tend to deviate from anticipated reaction conditions, making it difficult to determine its reaction characteristics clearly and accurately. In this regard, continuous-flow microreactors with unique advantages are considered to be effective tools for extracting reaction kinetic information, especially at high temperatures and pressures [18,19,20].

The typical procedure for preparing 1-aminoanthraquinone through ammonolysis reaction is to place 1-nitroanthraquinone in a batch reactor and treat it with aqueous ammonia, followed by several hours of stirring at 130 °C to 150 °C [21]. In order to convert 1-nitroanthraquinone within a reaction time of practical application value, a considerable amount of ammonia (the molar ratio of ammonia to 1-nitroanthraquinone 15~35) is necessary, which leads to an extremely high ammonia recovery load. Furthermore, ammonium nitrite produced during the ammonolysis process decomposes violently under high temperatures and pressures and may even explode. The 1-aminoanthraquinone preparation under continuous-flow mode in this study was conducted using N-methyl-2-pyrrolidone (NMP) as a solvent. With the process being maintained as safely as possible, a significant improvement in process efficiency was achieved. As part of our effort to determine whether continuous-flow technology can be advantageous for the preparation of 1-aminoanthraquinone, we conducted a series of experiments under continuous-flow mode, and the effects of a variety of reaction conditions on the preparation process (e.g., reaction temperature) were discussed in detail. By utilizing the Box–Behnken design in the response surface methodology, optimized reaction conditions were identified. By developing a kinetic model based on the mechanism of the ammonolysis reaction, an in-depth understanding of 1-aminoanthraquinone synthesis has been provided. 

## 2. Results and Discussion

### 2.1. Reaction Behavior Investigation

**Scheme 1:**
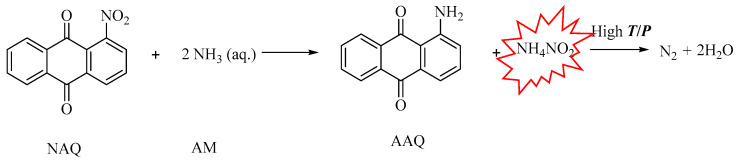
Reaction for the ammonolysis of NAQ with aqueous ammonia.

Generally, the ammonolysis of NAQ to prepare AAQ (Scheme 1) takes place in a batch reactor at high temperatures and pressures (high ***T***/***P***). However, ammonium nitrite will be produced during the ammonolysis process, which rapidly decomposes into nitrogen at high temperatures. As a result, the scale-up of the conventional batch ammonolysis process faces extremely challenging circumstances resulting from safety concerns. A continuous-flow strategy offers intrinsic safety characteristics that may contribute to the improvement of ammonolysis. In addition to the target reaction, the 1-aminoanthraquinone may undergo a secondary reaction to form 1-aminoanthraquinone imine, which is directly related to the reaction conditions [22]. Additionally, the intermolecular condensation of AAQ and the hydroxylation of NAQ all could occur during ammonolysis, resulting in the formation of byproducts [23,24].

#### 2.1.1. Influence of the Reaction Temperature

**Figure 1:**
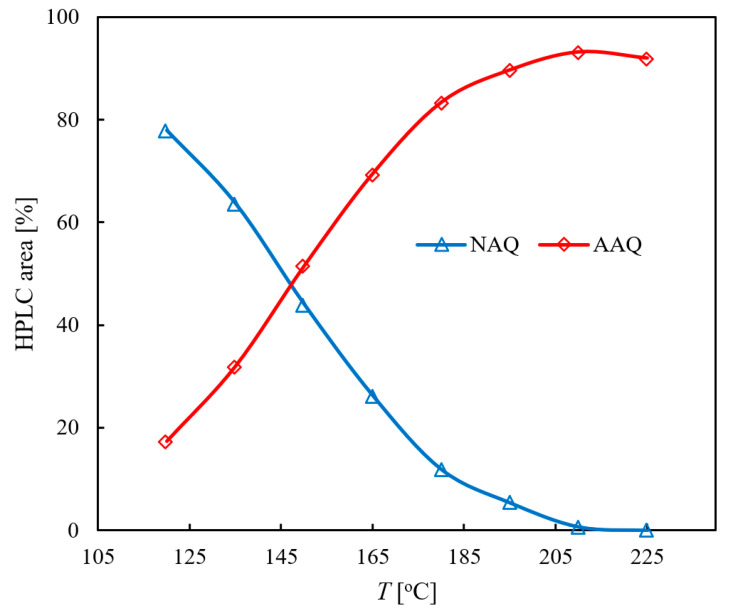
Influence of reaction temperature on the continuous-flow ammonolysis of NAQ. The concentration of NAQ solution = 0.13 M, *M-ratio* = 4.5, *RT* = 5 min, *T* = 120~225 °C.

In light of the fact that the conversion of NAQ to AAQ should be conducted at high temperatures to ensure that the ammonolysis process can be completed within an economical reaction time, temperatures ranging from 120 to 225 °C were screened at 1000 psi to identify the influence of reaction temperature on the continuous-flow ammonolysis of NAQ (Figure 1). As compared to batch operations, continuous-flow operations can provide a much safer and more reliable process under high temperatures and pressures. As the temperature increased, the reaction was able to be accelerated, but there was no continual increase in the level of AAQ formed. When the reaction temperature was increased from 120 to 225 °C, it was observed that the AAQ area percent rose from 17.4% to 93.2% and then fell back to 92.0%, possibly due to the fact that the side reactions were aggravated under high temperature. It was evident from the decrease in AAQ area percent at high temperature that it was necessary to accelerate the reaction rate at the appropriate temperature in order to optimize the selectivity of the AAQ.

#### 2.1.2. Influence of the Residence Time

**Figure 2:**
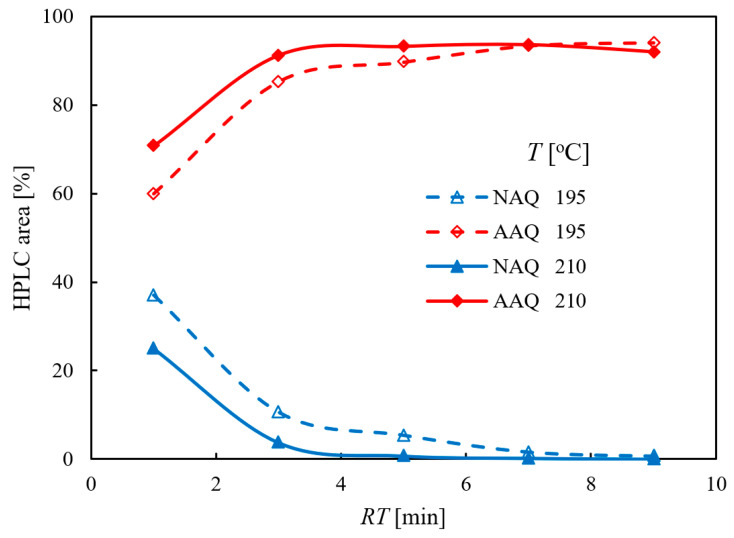
Influence of residence time on the continuous-flow ammonolysis of NAQ. The concentration of NAQ solution = 0.13 M, *M-ratio=* 4.5, *RT* = 1~9 min, *T* = 195 and 210 °C.

Figure 2 shows the relationship between residence time and the conversion of NAQ into AAQ under continuous-flow conditions. It was observed that as the residence time increased at 195 °C, the AAQ area percent increased quickly initially for the first three minutes, but then plateaued. As an example, in the initial 3.0 min, the AAQ area percent increased by 85.3%, while in the next 6 min, the AAQ area percent increased by only 8.8%. A sharp decrease in reaction rate was attributed to the decrease in NAQ concentration as residence time increased. Additionally, the AAQ area percent increased sharply and then decreased with increasing residence time from 1.0 min to 9.0 min at 210 °C, showing that choosing an appropriate residence time is crucial to achieve better AAQ formation at higher temperatures.

#### 2.1.3. Effect of the Water Content

**Figure 3:**
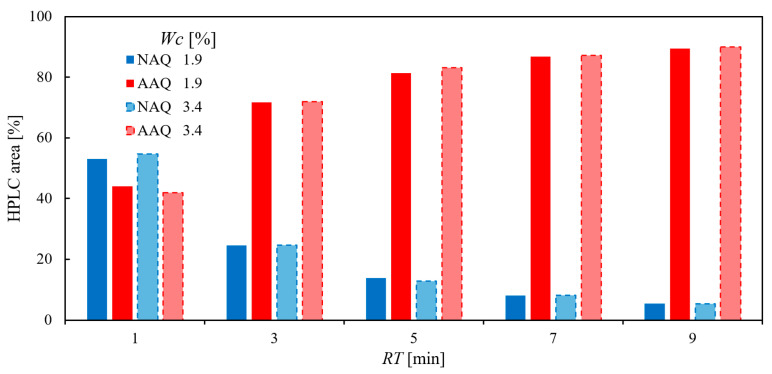
Effect of the *W_C_* on the continuous-flow ammonolysis of NAQ. The concentration of NAQ solution = 0.13 M, *M-ratio* = 3.0, RT = 1~9 min, *T* = 195 °C.

Aqueous solutions of ammonia are usually used in conventional experiments. The water is also a product during the decomposition of ammonium nitrite in our reaction process. Therefore, it was necessary to determine how water content (*W_C_*) affects the ammonolysis of NAQ. The water content, *W_C_*, is defined as the mass fraction of water in the reaction mixture. Neither conversion of NAQ nor the formation of AAQ were significantly affected by elevating *W_C_* in our experiments (Figure 3). In general, the conversion of NAQ or the formation of AAQ remained constant with minor fluctuations, which may explain the fact that the examined *W_C_* ranges were narrow, and the influence of *W_C_* on the ammonolysis reaction was not clearly evident. Due to the low solubility of anthraquinones in such conditions, it was not possible to evaluate the effects of higher *W_C_* on the reaction behavior.

#### 2.1.4. Screening of the *M-Ratio*

**Figure 4:**
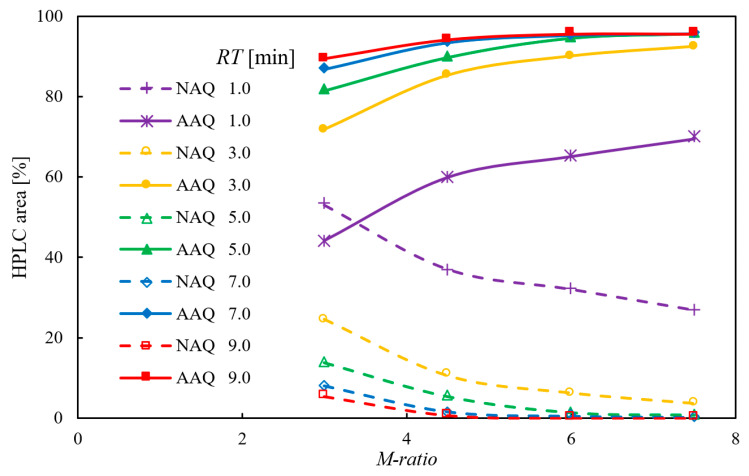
Screening of the *M-ratio* on the continuous-flow ammonolysis of NAQ. The concentration of NAQ solution = 0.13 M, *M-ratio* = 3.0~7.5, *RT* = 1.0~9.0 min, *T* = 195 °C.

*M-ratio*, defined as the molar ratio of ammonia to NAQ, was used to determine the amount of ammonia being used. The screening of the *M-ratio* ranging from 3.0 to 7.5 at various residence times (Figure 4) showed that the conversion of NAQ and the formation of AAQ could be effectively promoted initially, and then the promotion effect was significantly reduced. For example, a 15.8% increase on the AAQ area percent can be achieved by increasing the *M-ratio* from 3.0 to 4.5 at 1.0 min, but the AAQ area percent only increased by 9.6% as the *M-ratio* was increased from 4.5 to 7.5 at 1.0 min. Furthermore, the extreme excess of ammonia may result in the dramatic formation of 1-aminoanthraquinone imines [22]. Thus, comprehensively considering the recovery load of ammonia and its promoting effect on the reaction, a moderate excess of ammonia (e.g., an *M-ratio* of 3.0–5.0) was suitable for the preparation of AAQ.

### 2.2. Optimized Ammonolysis Process for the Preparation of AAQ

#### 2.2.1. Detailed Optimization Design of the Continuous Process

Among the many tools used to optimize the reaction process, the response surface methodology, or RSM, is one of the most widely used methods in this field [25,26]. In light of the previous investigation and discussion regarding the reaction behavior, this study was conducted using a Box–Behnken design with three levels and three factors. Through using the ranges and levels listed in Table 1, an experimental design matrix is provided in Table 2. The design suggests 17 experimental runs to be conducted under the concentration of NAQ of 0.13 M. The experimental error was determined by running runs 1, 9, 10, 14, and 17, which were the center points of the design. The experiments were conducted in accordance with the design, and the corresponding results are presented in Table 2. Analyzing the experimental results was performed using the response surface methodology based on a polynomial equation of the second order. Table 3 summarizes the results from the analysis of variance (ANOVA) conducted to evaluate the fitness and significance of the response surface quadratic model, the precision of the model, and the effects of variables and their interactions.

In view of the Model F-value of 474.97, it is evident that the model is significant. There is only a 0.01% probability that an F-value this large could occur due to noise. Significant model terms have *p*-values lower than 0.0500. The model terms A, B, C, AB, AC, BC, A^2^, B^2^, and C^2^ are all significant in the current case. F-value analyses indicate that residence time was the most influential factor in the formation of AAQ, followed by reaction temperature, and then molar ratio of ammonia to NAQ. Lack of fit F-value of 2.46 indicates that the lack of fit is not significant in relation to the pure error. There is a 20.28% chance that a lack of fit F-value this large could occur due to noise. Considering the predicted determination coefficient (*R*^2^) value of 0.9984, a mere 0.16 percent of the total variability cannot be explained by regressions and may be attributed to either human error or experimental error. The predicted *R*^2^ of 0.9821 and the adjusted *R*^2^ of 0.9963 are in reasonable agreement. Our ratio of 67.686 is significantly higher than 4, which indicates that this model displays an adequate signal and can thus be used to navigate the design space.

**Figure 5:**
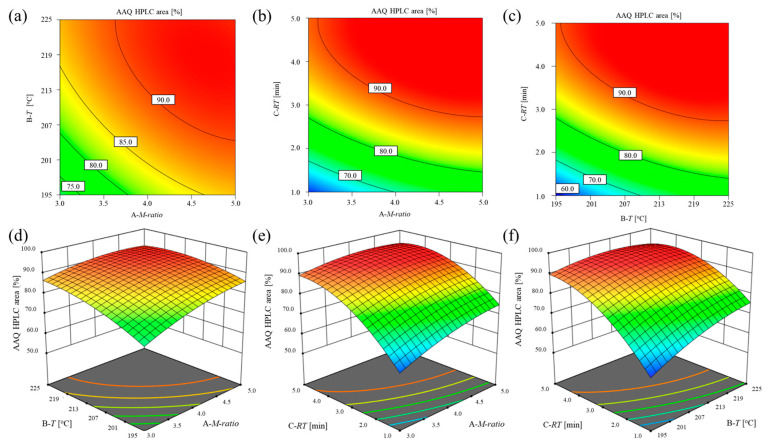
The influence of variable interaction on the formation of AAQ. (**a**–**c**) Contour plots. (**d**–**f**) Three-dimensional response surface plots.

An adequate understanding of how variable interaction affects AAQ formation can be gained by observing the response contour and the three-dimensional response surface plots, as shown in Figure 5. By using a two-dimensional contour representation of the three-dimensional surface (Figure 5a–c), it is possible to identify the key interactions that occur between the process variables based on the elliptical or circular property of the contours. A three-dimensional surface plot (Figure 5d–f) illustrates the extent to which responses vary when a single factor is considered and when a combination of two factors is considered. One of the primary goals of this study is to optimize the operation condition of the continuous-flow process to maximize the formation of AAQ. The optimized operation conditions within the established constraints for the preparation of AAQ were developed by the version 13 of Design Expert software. As a result of the above research, we have determined that the best conditions for formation of AAQ are the following: *M-ratio* of 4.5, reaction temperature of 213 °C, and residence time of 4.3 min.

#### 2.2.2. Optimized Process Reliability Evaluation

**Figure 6:**
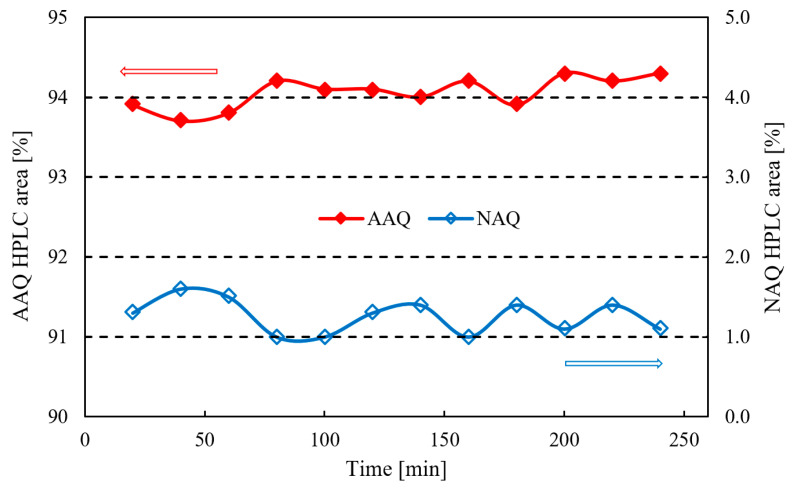
Process stability test under optimized conditions. The concentration of NAQ solution = 0.13 M, *M-ratio =* 4.5, *RT* = 4.3 min, *T* = 213 °C.

Through the application of the response surface methodology, we have previously identified the optimized reaction conditions for obtaining the maximum HPLC area percent of AAQ. Optimized process reliability was evaluated over a prolonged period of 4 h (Figure 6), and no blockages occurred in the reaction system. There were 12 samples collected for HPLC analysis, which were sampled at regular intervals of 20 min (>RT 4.3 min). According to the results obtained for these samples, the average HPLC area percent of AAQ could be recorded at 94.1%, while the average residual NAQ value was observed at 1.3%. As part of the optimization process, the yield of AAQ and the conversion of NAQ were obtained by collecting reaction mixtures from two different stages of 20 min and quantifying them using a standard internal method, with the yield and conversion being, respectively, ~88% and 98.4%. Table 4 summarizes the experimental data for the optimized process evaluation.

### 2.3. Kinetic Behavior Study for the Ammonolysis of NAQ

#### 2.3.1. Experimental and Kinetic Modeling Investigation

**Scheme 2:**
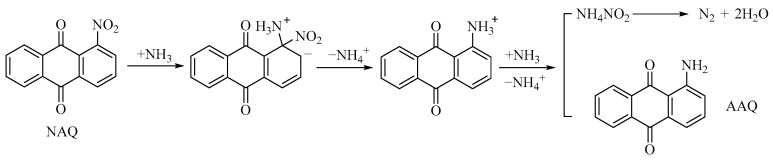
The evolution process for the ammonolysis of NAQ with ammonia.

The reaction between NAQ and ammonia follows a universally recognized ammonolysis mechanism [27]. The ammonolysis of NAQ under high temperature and pressure, however, is difficult to investigate accurately by kinetic experiments in conventional batch reactors. The exothermic property of the ammonolysis reaction and the volatile characteristics of ammonia will result in a wide temperature profile and serious deviation from the set molar ratio of ammonia to NAQ in the batch reactor. Despite these problems, the continuous-flow microreactor can deal with them in an effective manner. As part of the kinetic investigation, it is necessary to consider the interference from side reactions in the measurement of accurate kinetic data. Fewer than 7 percent impurities were detected during our kinetic experiments, which indicates that byproducts were not significant. The evolution process for the ammonolysis of NAQ with ammonia is presented in Scheme 2. Among the several reaction steps from NAQ to AAQ, the addition of NAQ with ammonia is usually considered to be considerably lower than that for the other reaction steps [21,27]. To sum up, only addition reaction between NAQ and ammonia was fully considered to analyze the data from kinetic experiments.

Hence, the apparent reaction rate for NAQ can be calculated as follows:

(1) $$-{dc}_{\mathrm{NAQ}}/{dt=k}_{\mathrm{app}}c_{\mathrm{NAQ}}c_{\mathrm{AM}}$$

where *k*_app_ represents the rate constant for the addition reaction between NAQ and ammonia. According to the law of conservation of mass, two molecules of AM will be consumed for every molecule of NAQ consumed. Therefore, a simple formula can be used to describe the concentration relation between NAQ and ammonia:

(2) $$c_{\mathrm{AM}}{=2c}_{\mathrm{NAQ}}{+c}_{AM0}-{\;2c}_{NAQ0}$$

where $c_{AM0}$ and $c_{NAQ0}$ represent the initial AM and NAQ concentrations in the mixed reaction raw material, respectively. Combining the equations mentioned above allows the reaction rate for NAQ to be expressed as follows:

(3) $$-{dc}_{\mathrm{NAQ}}/{dt=k}_{\mathrm{app}}{(2c}_{\mathrm{NAQ}}{+c}_{AM0\;}-{\;2c}_{NAQ0}{)c}_{\mathrm{NAQ}}$$

The deformed reaction rate equation of NAQ can be integrated from $c_{NAQ0}$ at 0 min to $c_{\mathrm{NAQ}}$ at *t* min, and the evolution of the concentration of NAQ over residence time can be written as

(4) $${\ln\;[2c}_{\mathrm{NAQ}}/\left(c_{AM0}-{\;2+2c}_{\mathrm{NAQ}}\right)]\;{=(2c}_{NAQ0\;}-{\;c}_{AM0}{)k}_{\mathrm{app}}t+\ln\left(2c_{NAQ0}/c_{AM0}\right)$$

**Figure 7:**
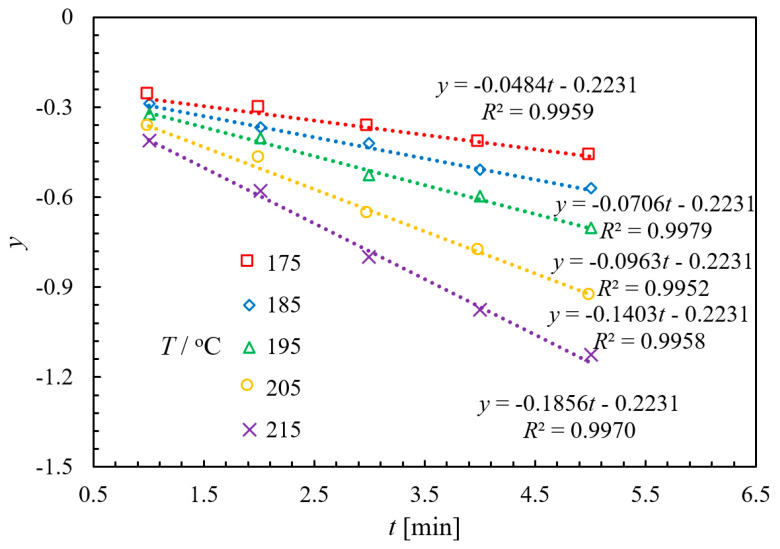
Linear relationship between *y* (${\ln\;[2c}_{\mathrm{NAQ}}/\left(c_{AM0}{\;-\;2c}_{NAQ0}{+2c}_{\mathrm{NAQ}}\right)]$) and *t* at different temperatures. The concentration of NAQ solution = 0.13 M, *M-ratio* = 2.5, *RT* = 1.0~5.0 min, *T* = 175~215 °C.

The linear relationship between ${\ln\;[2c}_{\mathrm{NAQ}}/\left(c_{AM0}-{\;2c}_{NAQ0}{+2c}_{\mathrm{NAQ}}\right)]$ (*y*) and *t* at different temperatures can be depicted using the data from the kinetic experiments (Figure 7). Afterwards, the apparent reaction rate constant *k*_app_ can be deduced and summarized as shown in Table 5.

The relationship between apparent reaction rate constant and reaction temperature can be expressed by the classical Arrhenius equation, which is transformed into another form (Equation (5)) to obtain kinetic parameters by using simple linear regression.

(5) $${\ln k}_{\mathrm{app}}=-E_{\mathrm{app}}{/(\mathrm{RT})+\mathrm{lnA}}_{\mathrm{app}}$$

where *A*_app_ represents apparent pre-exponential factor (L·mol^−1^·min^−1^) and *E*_app_ is the apparent activation energy (kJ·mol^−1^).

**Figure 8:**
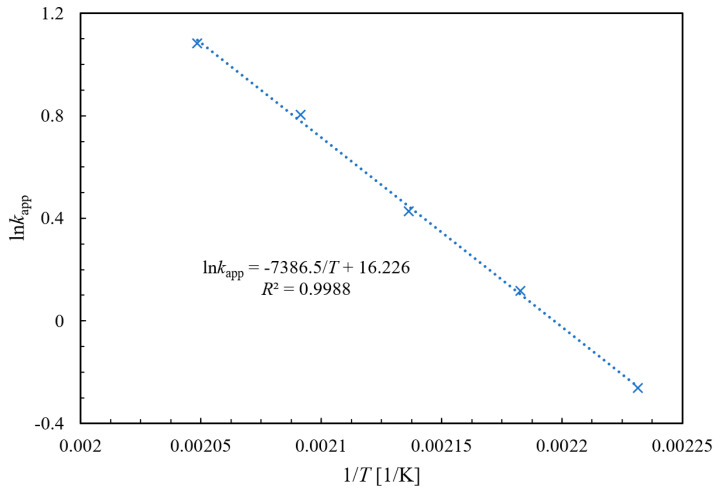
Linear relationship between ln*k*_app_ and 1/*T*.

The linear relationship between ln*k*_obs_ and 1/*T* is shown in Figure 8. Through the linear fitting, the apparent activation energy *E*_app_ and pre-exponential factor *A*_app_ can be calculated to be 61.41 kJ/mol and 1.11 × 10^7^ L·mol^−1^·min^−1^, respectively.

#### 2.3.2. Validation Verification of Kinetic Experiments

The reaction kinetics of ammonolysis of NAQ is discussed based on the fact that the reaction system is operated under isothermal conditions, while it is extremely difficult to achieve absolute isotherms in an exothermic reaction. During an exothermic reaction in a continuous-flow microreactor, a maximum temperature point will arise in the axial direction of the reactor, causing the reaction process to deviate from the set reaction temperature and resulting in inaccurate experimental kinetic data. To verify the reliability of kinetic experimental data, it is necessary to look at the distribution of axial temperature in the continuous-flow microreactor. Establishing a reasonable equation to describe the axial temperature distribution in the continuous-flow microreactor is the first step [28,29]. Because all kinetic experimental data are obtained under steady-state conditions, the mass and heat transfer equations employed in the continuous-flow microreactor are time-independent. The corresponding mass balance law can be simplified deformed as follows.

(6) $$D_{\mathrm{ax}}\frac{d^{2}c_{\mathrm{NAQ}}}{{dz}^{2}}-\;u\frac{{dc}_{\mathrm{NAQ}}}{dz}{+v}_{\mathrm{NAQ}}r_{\mathrm{NAQ}\;}=0$$

where *D*_ax_ is axial dispersion coefficient, *z* is channel length coordinate, and *u* is flow velocity. *D*_ax_ for laminar flow in a cylindrical channel can be estimated by the correlation of Taylor and Aris [30,31]:

(7) $$D_{\mathrm{ax}}{=D}_{m}+\frac{u^{2}d_{i}^{2}}{{192D}_{m}}$$

where $D_{m}$ is the molecular diffusion coefficient and *d*_i_ is the inner tube diameter. For the heat transfer behavior in a continuous-flow microreactor, assuming that the heat capacity and reaction enthalpy during the ammonolysis of NAQ are constant, the following energy balance equation can be obtained with no axial work and no changes in kinetic energy and potential energy.

(8) $$A_{\mathrm{ax}}\frac{d^{2}T}{{dz}^{2}}-\;u\frac{dT}{dz}-\frac{r_{\mathrm{NAQ}}{\Delta H}_{r}}{{\rho c}_{p}}-\frac{\alpha_{i}}{{\rho c}_{p}}(T\;-{\;T}_{o})\frac{4}{d_{i}}=0$$

where *A*_ax_ is thermal dispersion coefficient, $r_{\mathrm{NAQ}}$ is the reaction rate of NAQ, $\alpha_{i}$ is heat transfer coefficient, $c_{p}$ is specific heat capacity, $d_{i}$ is the inner tube diameter, and $T_{o}$ is the temperature of fluid outside the tube. The heat transfer process in the continuous-flow microreactor involves the heat transfer process of cold and hot fluids on both sides of the reactor and the heat conduction process of the solid wall. Therefore, the calculation of the heat transfer coefficient $\alpha_{i}$ must include the above factors. In terms of thermal resistance, heat transfer coefficient can be calculated as follows.

(9) $$\frac{1}{\alpha_{i}}=\frac{1}{h_{i}}+\frac{{\delta d}_{i}}{{\lambda d}_{m}}+\frac{d_{i}}{h_{o}d_{o}}$$

where $h_{i}$ is the heat transfer coefficient of inner fluid, $h_{o}$ is the heat transfer coefficient of outer fluid, δ is the wall thickness, λ is the thermal conductivity of wall, $d_{o}$ is the outer diameter of tubular microreactor, and $d_{m}$ is the arithmetic average diameter of the tubular microreactor, which can be derived as follows.

(10) $$d_{m}=\frac{d_{o}-d_{i}}{{\ln(d}_{o}{/d}_{i})}$$

Turbulent fluid heats or cools the outer side of the tubular microreactor, and the outer fluid’s heat transfer resistance is negligible compared to the wall’s and the inner side’s. Hence, the outer fluid’s heat transfer resistance will not be considered. In all cases, $h_{i}$ was defined by assuming fully developed laminar-flow heat transfer with *Nu* = 3.66 (constant wall temperature) [32]. Physical properties parameters, such as thermal conductivity of reaction fluid k and specific heat capacity, are related to reaction temperature, and they can be obtained from chemical properties handbooks [33,34]. As enthalpy is a crucial component of the calculation of heat balance, the classical group contribution method can be used to estimate the reaction enthalpy ${\;\Delta H}_{r}$ [35,36,37,38].

In Equations (6) and (8), the second-order derivative terms describe the conduction of heat and diffusion of mass axially. The existence of second-order derivative terms in the equations increases the complexity of the numerical solution of the equation, while the influence of second-order derivative terms on the solution results is not significant because the reactor length is long enough compared to the inner diameter of the reactor (*L*/*d*_i_ > 10,000). For example, the axial diffusion behavior in the tubular reactor can be neglected when the *Bo* number is greater than 100, and the calculated *Bo* number is in the range of 348~2735 in our kinetic experiments [39,40]. The value of the *Bo* number was obtained by multiplying flow velocity *u* by reactor length *L* and dividing by axial dispersion coefficient *D*_ax_. Thus, the axial heat conduction and axial diffusion behavior in the reaction process were neglected to provide a simplified transfer model (STM), as follows.

(11) $$-u\frac{{dc}_{\mathrm{NAQ}}}{\partial z}{+v}_{\mathrm{NAQ}}r_{\mathrm{NAQ}}=0$$

(12) $$-u\frac{dT}{dz}-\frac{r_{\mathrm{NAQ}}{\Delta H}_{r}}{{\rho c}_{p}}-\frac{\alpha_{i}}{{\rho c}_{p}}(T\;-{\;T}_{o})\frac{4}{d_{i}}=0$$

The first derivative designates the axial transport by convection and the remaining terms are the source terms. In this case, the source terms are the heat exchanged with the neighboring wall and the transformation/heat formed by the reaction. During the analysis of kinetic experiment data, the flow behavior in the continuous-flow microreactor was considered as isothermal plug flow behavior; therefore, the simplified transfer model was further transformed into an ideal plug flow model (IPF), as follows:

(13) $$-u\frac{{dc}_{\mathrm{NAQ}}}{dz}{+v}_{\mathrm{NAQ}}r_{\mathrm{NAQ}}=0$$

(14) $$-u\frac{dT}{dz}=0$$

**Figure 9:**
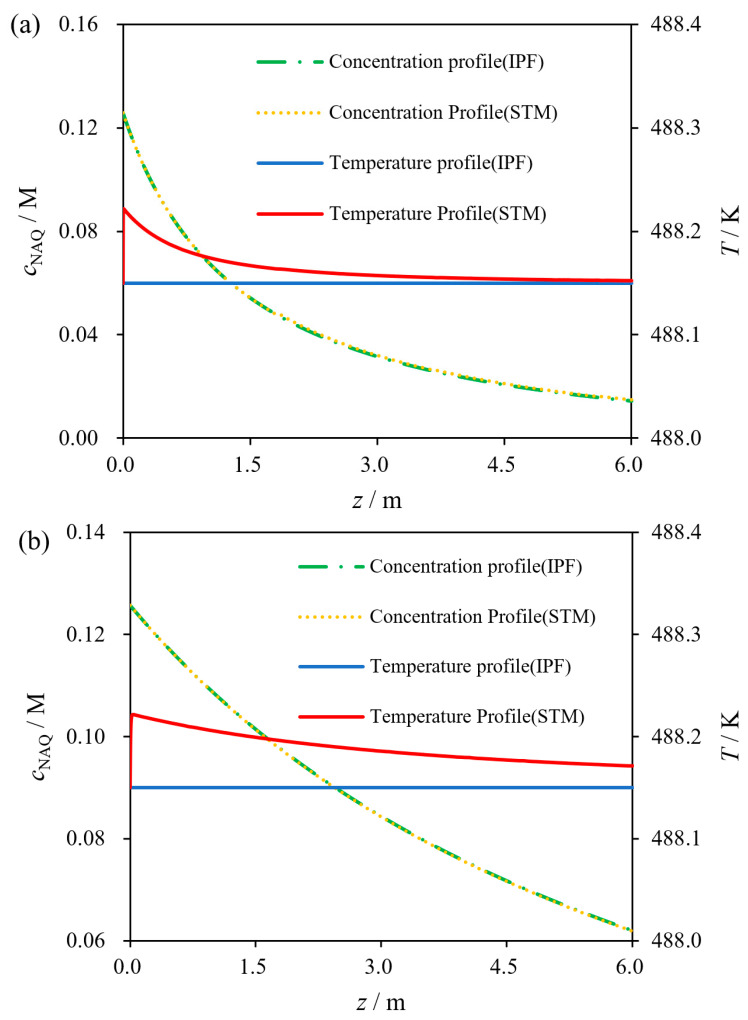
The predicted concentration and temperature profiles versus axial distance *z* between different transfer models. (**a**) The concentration of NAQ solution = 0.13 M, *M-ratio =* 2.5, *RT* = 5.0 min, *T* = 215 °C. (**b**) The concentration of NAQ solution = 0.13 M, *M-ratio =* 2.5, *RT* = 1.0 min, *T* = 215 °C.

To describe the axial concentration and temperature profiles of continuous-flow ammonolysis under steady-state conditions, simplified transfer models and ideal plug flow models were both implemented. As shown in Figure 9a,b, the maximum temperature rise along the axial distance *z* during the exothermic ammonolysis reaction is less than 0.1 K, even at the highest reaction temperature of 488.15 K. Meanwhile, the predicted concentration evolution with axial distance z is almost consistent for both models, confirming the reliability of continuous-flow microreactors in extracting reaction kinetic information, and the ideal isothermal plug flow model can be used to obtain key kinetic parameters.

**Figure 10:**
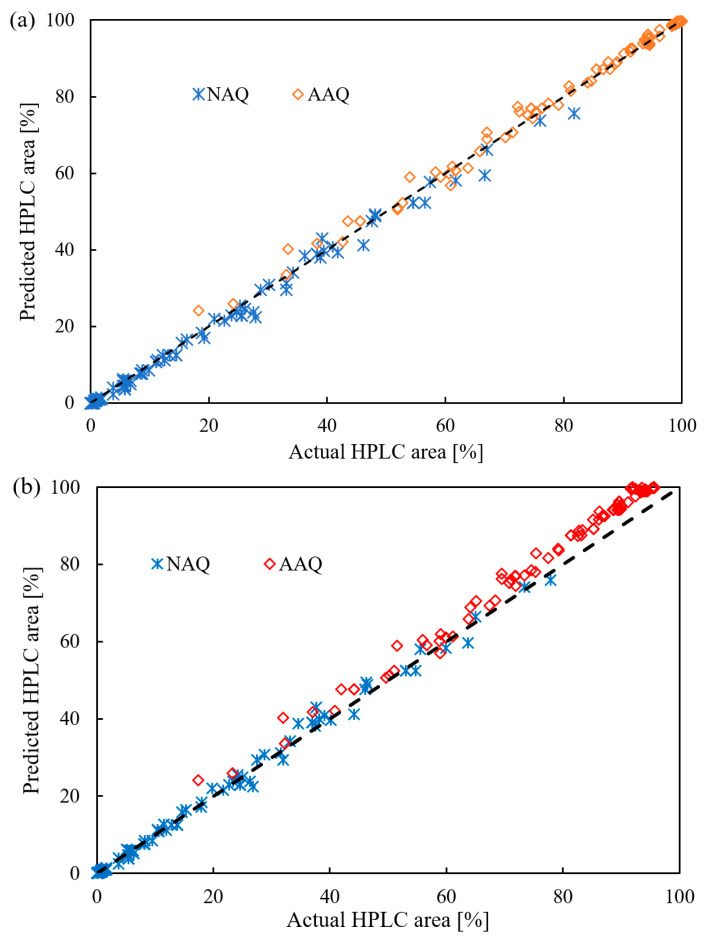
Comparison of experimental and predicted conversion of NAQ and formation of AAQ. (**a**) Side reactions were ignored. (**b**) Side reactions were taken into consideration.

The kinetic model was further employed to predict the conversion of NAQ and the formation of AAQ under a variety of conditions (reaction temperature, residence time, molar ratio of ammonia to NAQ, and water content) for the purpose of assessing the applicability of the model. A comparison was made between the observed values of NAQ and AAQ HPLC area percent and those predicted by the above kinetic model, as depicted in Figure 10. The difference between Figure 10a,b is whether the side reactions were ignored. These two values for NAQ and AAQ are very close in Figure 10a. As a result of the consideration of side reactions in Figure 10b, the comparison does not exhibit excellent agreement as it does in Figure 10a; however, the experimental results are still consistent with the predictions made by the model, indicating that the kinetic model may still be able to accurately describe AAQ synthesis even if side reactions are taken into account.

## 3. Experimental Section

**Figure 11:**
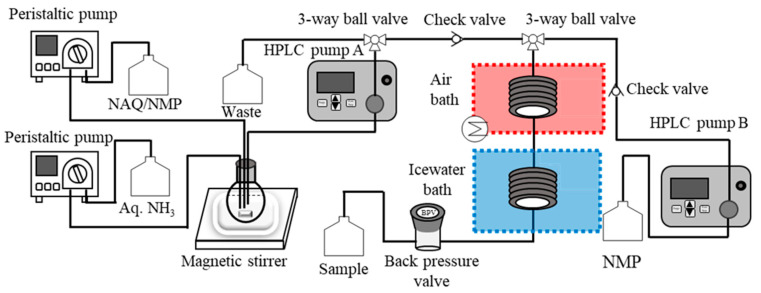
The configuration of the continuous-flow system for synthesizing AAQ through ammonolysis reaction.

The chemical reagents used in the experiment were all purchased commercially and were not individually purified before use. The water used in our laboratory was deionized and employed throughout the process. By screening a wide range of conventional solvents, N-methyl-2-pyrrolidone (NMP) was found to be a relatively good solvent for dissolving 1-nitroanthraquinone (NAQ) and 1-aminoanthraquinone (AAQ) due to their poor solubility in a variety of solvents. Figure 11 provides a schematic illustration of the continuous-flow ammonolysis system for producing AAQ. The massive use of NMP enabled the ammonolysis reaction of NAQ to be converted into a continuous-flow mode because handling solids in a continuous-flow microreactor is still challenging. The schematic in Figure 11 shows the configuration of the continuous-flow system for synthesizing AAQ through ammonolysis reaction.

As shown in Figure 11, NAQ solution and ammonia solution were pre-mixed in a round bottom flask, in which the convenient mixing manner through a Y-shape or T-shaped mixer was not adopted due to the large flow difference between the two feeding streams. Magnetic stirring was used to mix a NAQ solution and an ammonia solution, which were delivered by BT100-1F peristaltic pumps, and then a mixture of raw materials was produced. A 316 L stainless steel flow channel with an internal diameter of 0.6 mm was constructed, and the mixture was introduced into the channel by Series III HPLC pump A. Check valves were incorporated into the reaction system to prevent reverse flow of the reactants during the reaction. Generally, the entire flow channel was divided into two zones, including the reaction zone and the inhibition zone. The flow channel in the reaction zone was submerged in the high-temperature air bath to ensure precise control of the reaction temperature. This reaction was inhibited by immersing the flow channel of the inhibition zone in an ice–water bath. Installing a back-pressure valve with a fixed pressure of 1000 psi at the end of the flow channel can stabilize the superheated reaction system. In addition, the high operating pressure can compress the nitrogen generated during the reaction (~1/70 volume at atmospheric pressure) to prevent its impact on the residence time and process stability. Residence time inside the flow channel can be accurately controlled by altering the flow velocity. For a given flow velocity, the samples were collected for analysis after waiting continuously for a minimum of three times the mean residence time to establish a steady state under the set experimental conditions. The 3-way ball valve adjacent to the HPLC pump B can be switched to deliver the solvent NMP into the reactor channel in order to check the tightness of the system or to clean the reactor system when the process conditions are altered or the channels are blocked. In the event that the process conditions change, switching the 3-way ball valve adjacent to the HPLC pump A may be necessary to remove the residual raw material mixture.

In order to determine the peak area percent of NAQ, AAQ, as well as byproducts contained in the quenched samples, HPLC analysis was performed on the samples. For the determination of the conversion of NAQ and yield of AAQ, we used an internal standard method that consisted of adding nitrobenzene to the sample in the form of an internal standard to serve as a reference for determining the conversion of NAQ and yield of AAQ. Analyses were performed using an Eclassical 3100 high-performance liquid chromatography equipped with an Agilent EC-C18 column (150 mm × 4.6 mm, particle size 5 mm). The mobile phase consisted of water containing 0.05% TFA (A) and methanol (B) at a flow rate of 1.2 mL/min. The gradient elution program was as follows: 0–9 min, 70–30% A; 9–11 min, 30–30% A; 11–11.1 min, 30–70% A; 11.1–16 min, 70–70% A. The temperature of the column and the wavelength of detection were set at 30 °C and 254 nm, respectively.

## 4. Conclusions

Through the ammonolysis of 1-nitroanthraquinone with aqueous ammonia in a continuous-flow microreactor, a method possessing the advantages of high controllability and intrinsic safety was developed to synthesize 1-aminoanthraquinone efficiently. A detailed investigation of the effect of reaction conditions, including reaction temperature, residence time, and the molar ratio of ammonia to 1-nitroanthraquinone on the continuous-flow preparation of 1-aminoanthraquinone has been carried out. The optimized operation condition for the preparation of 1-aminoanthraquinone was developed through a classic Box–Behnken design to achieve effective conversion of 1-nitroanthraquinone, in which the yield of 1-aminoanthraquinone and the conversion of 1-nitroanthraquinone are 34~88% and 98.4%, respectively. The optimized continuous-flow process’s reliability was evaluated through a 4 h continuous-flow experiment. To optimize the reactor design and to gain a deeper understanding of the ammonolysis process, the key kinetic parameters for the preparation of 1-aminoanthraquinone were obtained under continuous-flow mode.

## Figures and Tables

**Table 1:** Independent variables and their levels and level for an experimental design.

Level	Coded Level	Uncoded Level		
		A: *M-Ratio*	B: *T* [°C]	C: *RT* [min]
low	−1	3	195	1
mid	0	4	210	3
high	1	5	225	5

**Table 2:** Experimental design matrix and corresponding results for the preparation of AAQ.

Run	Actual Level of Variables			AAQ HPLC Area [%]	
	*M-Ratio*	*T* [°C]	*RT* [min]	Observed	Predicted
1	4.0	210	3.0	88.8	89.2
2	5.0	210	5.0	92.5	93.1
3	4.0	225	5.0	91.7	91.8
4	3.0	210	1.0	61.1	60.6
5	4.0	225	1.0	75.4	75.8
6	3.0	195	3.0	71.8	72.5
7	3.0	210	5.0	89.6	89.3
8	5.0	225	3.0	91.7	91.1
9	4.0	210	3.0	89.6	89.2
10	4.0	210	3.0	89.8	89.2
11	5.0	195	3.0	86.2	86.0
12	4.0	195	1.0	58.9	58.8
13	3.0	225	3.0	86.3	86.5
14	4.0	210	3.0	89.4	89.2
15	4.0	195	5.0	90.0	89.7
16	5.0	210	1.0	74.6	74.9
17	4.0	210	3.0	88.6	89.2

**Table 3:** An analysis of variance for a quadratic model of response surfaces.

Source	Sum of Squares	df	Mean Square	F-Value	*p*-Value	
Model	1860.74	9	206.75	474.97	<0.0001	significant
A-*M-ratio*	163.81	1	163.81	376.32	<0.0001	
B-*T*	182.40	1	182.40	419.05	<0.0001	
C-*RT*	1099.81	1	1099.81	2526.63	<0.0001	
AB	20.25	1	20.25	46.52	0.0002	
AC	28.09	1	28.09	64.53	<0.0001	
BC	54.76	1	54.76	125.80	<0.0001	
A^2^	24.15	1	24.15	55.48	0.0001	
B^2^	34.08	1	34.08	78.29	<0.0001	
C^2^	230.26	1	230.26	528.98	<0.0001	
Residual	3.05	7	0.4353			
Lack of Fit	1.98	3	0.6583	2.46	0.2028	not significant
Pure Error	1.07	4	0.2680			
Cor Total	1863.79	16				

**Table 4:** Experimental data for the optimized process evaluation.

Operation Conditions	Results						
	HPLC Area			Stage 1		Stage 2	
*M-ratio* = 4.5 *T* = 213 °C *RT* = 4.3 min	Experimental NAQ	Experimental AAQ	Predicted AAQ	Conversion of NAQ	Yield of AAQ	Conversion of NAQ	Yield of AAQ
	1.3% (Average)	94.1% (Average)	94.7%	98.4%	88.3%	98.4%	87.9%

**Table 5:** Apparent reaction rate constant for the ammonolysis of NAQ at various temperatures.

*T*/°C	175	185	195	205	215
*k*_app_/L·mol^−1^·min^−1^	0.7701	1.1233	1.5322	2.2323	2.9531

## Data Availability

Not applicable.
